# Indoor-Outdoor Detection Using a Smart Phone Sensor

**DOI:** 10.3390/s16101563

**Published:** 2016-09-22

**Authors:** Weiping Wang, Qiang Chang, Qun Li, Zesen Shi, Wei Chen

**Affiliations:** College of Information Systems and Management, National University of Defense Technology, Changsha 410073, China; wang.wp2010@gmail.com (W.W.); liqun@nudt.edu.cn (Q.L.); nudtshizesen@126.com (Z.S.); weichen@nudt.edu.cn (W.C.)

**Keywords:** seamless positioning, indoor/outdoor detection, machine learning, GSM

## Abstract

In the era of mobile internet, Location Based Services (LBS) have developed dramatically. Seamless Indoor and Outdoor Navigation and Localization (SNAL) has attracted a lot of attention. No single positioning technology was capable of meeting the various positioning requirements in different environments. Selecting different positioning techniques for different environments is an alternative method. Detecting the users’ current environment is crucial for this technique. In this paper, we proposed to detect the indoor/outdoor environment automatically without high energy consumption. The basic idea was simple: we applied a machine learning algorithm to classify the neighboring Global System for Mobile (GSM) communication cellular base station’s signal strength in different environments, and identified the users’ current context by signal pattern recognition. We tested the algorithm in four different environments. The results showed that the proposed algorithm was capable of identifying open outdoors, semi-outdoors, light indoors and deep indoors environments with 100% accuracy using the signal strength of four nearby GSM stations. The required hardware and signal are widely available in our daily lives, implying its high compatibility and availability.

## 1. Introduction

In the era of mobile internet, we can connect to the internet any place and any time with portable devices. As a result, Location Based Services (LBS) have developed dramatically. The integration of traditional services and location information has brought about a great deal of innovative applications. All of these LBS applications have the same requirement: the user’s current position.

The users may by in any place, such as in the open outdoors, crowded avenues, deep indoors and so on. The next generation of positioning systems must work well in a range of environments to meet the needs of a variety of LBS applications. Researchers have developed many positioning technologies for different environments. No single positioning technology is robust enough to perform well in all of these environments. However, there is an accurate enough positioning technique for any specific environment. For example, Global Navigation Satellite Systems (GNSS) performs well in open sky environments, while Shadow Matching [1] is enough for urban canyon environments, and Wi-Fi fingerprint positioning is suitable for indoor environments. This implies that we can integrate all these positioning techniques for Seamless indoor and outdoor Navigation and Localization (SNAL) applications [2]. However, the battery power is a limitation for any portable smart device. Turning on all of the required sensors is energy consuming. Letting the user manually choose different techniques according to different environments is not user friendly. Detecting the users’ current environments automatically and efficiently with low battery consumption is crucial for SNAL. In this paper, we focus on the automatic indoor/outdoor detection problem in SNAL.

There are a lot of previous works focused on indoor/outdoor detection to provide essential information for upper layer applications [3]. They mainly work in two types of environment: indoor and outdoor environments [4], but for SNAL, these two environments are not enough. In this paper, we focused on four types of environments: open outdoors, semi-outdoors, light indoors and deep indoors. These environments are shown in Table 1.

In the open outdoors environment, at least four satellites are available for positioning because of the open sky condition. Semi-outdoors represents a GNSS-hostile outdoor environment such as an urban canyon or a wooded area, where there are not enough satellites for positioning. Light indoors is similar to a semi-outdoors environment. Deep indoors environment refers to a place with no satellite in view.

In each of these four environments, there was a well-developed positioning algorithm for localization. In the open outdoors environment, no matter whether on water or on a highway, we can localize ourselves using GNSS [5]. In the semi-outdoors environment, urban canyons and wooded areas are both capable of using GNSS shadow matching for positioning [1]. In the light indoors environment, cooperative positioning was suitable [6], while in deep indoors environments, a fingerprint positioning algorithm is the most commonly used technique [7,8].

The difference between open outdoors and semi-outdoors is the number of satellites in view. In open outdoor environments, at least four satellites are available, which means the user can be localized by GNSS, while in semi-outdoors environments, the number of visible satellites is not enough for GNSS localization. The difference between light indoors and deep indoors is the visibility of navigation satellites. In light indoors environments, users may receive navigation signals from several satellites. As a result, the users in light indoor environments can be localized using peer to peer cooperative positioning, but in deep indoor environments, no navigation satellites are available. In that case, cooperative positioning fails.

Many works address the issue of detecting indoor/outdoor environments. Walter et al. identified a set of novel environmental features that could be used for environment detection [9]. These features included gravity, ambient light, magnetic fields, scents, road signs, temperature, terrain height, road texture and so on. Some of the related works focused on environment sensing using mobile device’s sensors based on these features. The sensors used included Global Positioning System (GPS), accelerometers, gyroscopes, barometers, geomagnetic sensors, Wi-Fi cards and so on. In Groves’s work, environmental context detection using GNSS and Wi-Fi were examined [10]. The results showed that GNSS C/No measurement can be used to distinguish indoor from outdoor environments and to distinguish different types of outdoor environment, such as urban and open. Wi-Fi measurements have been shown to be unreliable for distinguishing betweenindoor and outdoor environments, but good for distinguishing different outdoor types, such as residential and business districts. Muralidharan proposed to use a barometer and GPS to identify different floors in indoor environments [11]. Ravindranath et al. showed that GPS lock status can be used to indirectly infer the ambient environment [12]. The GPS, however, consumed too much energy to be useful for many applications. A smart phone may run out of energy in about 6 h if the GPS is running continuously [13], and furthermore, GPS was only available in open sky environments [14]. As a result, researchers proposed to use the low energy consumption sensors available in smart phones for detecting the environment, such as accelerometers, gyroscopes, barometers and magnetic sensors. Mostafa et al. made use of accelerometers, gyroscopes, barometers, and magnetic sensors to detect the height changing modes of the user in indoor environments [15,16,17]. However, accelerometers are orientation and position-dependent, a require a high sampling rate to achieve good accuracy. Researchers sought to use alternative sensors. For example, IODetector detected indoors, outdoors and semi-outdoors environments using cell signals, light and magnetic intensity [2]. Vanini proposed to use barometers to detect the change of floors [18]. TempIO classified the indoor/outdoor environment by comparing the temperature [19]. Wu et al. showed that barometers were capable of monitoring door events [20]. Barometer and temperature sensors are not widely available in current mobile phones. Radu et al. presented a general method employing semi-supervised machine learning and used light intensity, cellular signal strength, magnetic variance and sound intensity [3]. They provided a detection accuracy exceeding 90%, but their algorithm relied on several sensors, which is not energy efficient. Detecting indoor/outdoor environments using widely available sensors with low energy consumption remains a challenge for researchers.

Our research was motivated by these pioneering works, but we went further. We proposed to use the GSM signal strength to detect four types of indoor/outdoor environments. Firstly, GSM is available on all GSM-based smart phones. Secondly, it consumes minimal energy in addition to standard cell-phone operation [21]. The basic idea was simple: the propagation of radio signals is affected by the environment. Different environments result in different signal strength characteristics. By identifying the signal strength’s characteristics, we can determine the user’s environment. We have investigated a wide range of machine learning algorithms for classification, including Decision Tree (DT), Random Forest (RF), Support Vector Machine (SVM), K Nearest Neighbor (KNN), Logistic Regression (LR), Naive Bayesian (NB) [22], and Neural Network (NN). The classifiers were applied for recognition.

Experimental results showed that the proposed algorithm was capable of detecting open outdoors, semi-outdoors, light indoors and deep indoors environments with 100% accuracy using four nearby GSM stations’ signal strength. The required hardware and signals are widely available in our daily lives, implying its high compatibility and availability.

## 2. Methodology

**Figure 1:**
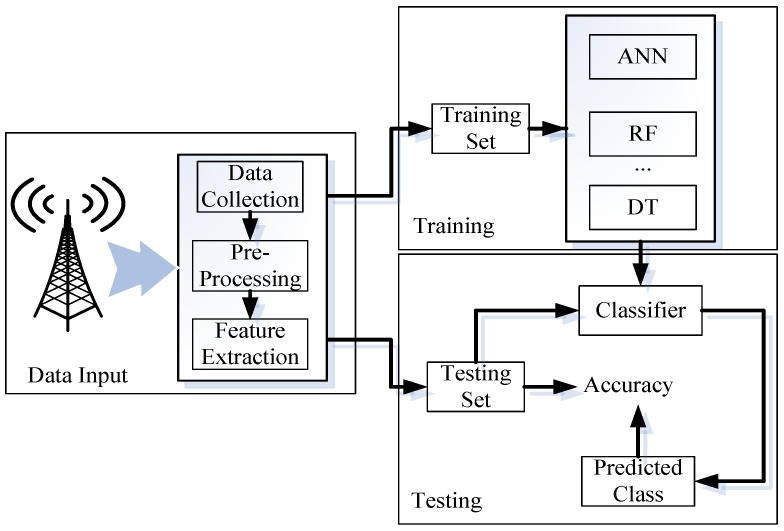
Framework of indoor/outdoor detection. This framework contains three processes: Data Input, Training, and Testing. In the Data Input process, neighboring cellular base stations’ signal strength is recorded, and features are extracted. The data are classified in the Training phase. The classifier is applied to detect the user’s current environment in the Testing phase.

The proposed algorithm contains three processes: Data Input, Training, and Testing. An overview of the indoor/outdoor detection process is illustrated in Figure 1.

### 2.1. Data Input

In the Data Input process, three sub-processes are included: Data Collection, Pre-Processing, and Feature Extraction.

#### 2.1.1. Data Collection

In this process, GSM signal strength is recorded in different environments. According to the GSM standard, each smart phone records a vector of the detected power levels of the pilot signal from at most seven cellular base stations, one of which the smart phone is associated with [21]. For the Android Operating System, the received signal strength index (RSSI) for GSM is in asu, where 0 asu means −113 dBm and 31 asu means −51 dBm. So we have:

(1) rss=−113+2*rssi

The recorded signal strength is arranged from the strongest to the weakest. Assuming that the sampling interval is *τ* (s), the sampling frequency is 1/*τ* Hz. The data collected during *T*(s) is denoted as *S*, where:

(2) $$S=\{s_{i}|i=0,1,\cdots ,\left\lfloor T/\tau \right\rfloor \},\;s_{i}=\{rss_{i,1},rss_{i,2},\cdots ,rss_{i,n_{i}}\},rss_{i,m}\geq rss_{i,n}\forall \text{m}>\text{n}$$

where *n_i_* is the number of visible stations at time *i*.

#### 2.1.2. Data Pre-Processing

The environment is changing continuously, therefore, we need to look at how a signal changes over a specific period of time to identify the environment. A window is the most basic step and is used by almost all researchers. At each time epoch, the signal involving the current measurement and the previous *N* − 1 epochs, where *N* is a chosen integer that represents the length of the window. Assuming the window length is ∆*T*(s), so we have *N* = ⌊Δ*T*/*τ*⌋, the measurement is arranged as:

(3) $$W=\{w_{i}|i=0,1,\cdots ,\left\lfloor T/△T\right\rfloor \},\mathrm{where}\;w_{t}=\{s_{t-i}|i=0,1,\cdots ,\left\lfloor \Delta T/\tau \right\rfloor -1\}$$

Different window lengths contain different information used for detection. The wider the window is, the more descriptive the features extracted from it can be. While the shorter the window, the less information it contains for detection.

#### 2.1.3. Feature Extraction

In each window, we can extract several features to describe the character of the environment. These features include Mean, Standard Deviation, Maximum, Minimum and Range.

(1) Mean
  Mean is the most basic character of a signal. It is calculated by summing the values and dividing the numbers:
  (4) $$mean(w_{t})=\sum w_{t}/|w_{t}|$$
  where |*| is the number getting operation. Mean is a measure of the middle value of a signal.
(2) Standard Deviation
  Standard Deviation is an indicator of how much a signal is dispersed around its mean. It is calculated as follows:
  (5) $$Std(w_{t})=\sqrt{\sum {\left(s_{i}-\mathrm{mean}(w_{t})\right)}^{2}/|w_{t}|}$$
(3) Maximum and Minimum
  Maximum and Minimum values are the extreme values in the window:
  (6) $$Max(w_{t})=s_{i}\in w_{t},\forall s_{j}\in w_{t},s_{j}<s_{i},Min(w_{t})=s_{i}\in w_{t},\forall s_{j}\in w_{t},s_{j}>s_{i}$$
(4) Range
  Range is the difference between the Maximum and Minimum values:
  (7) $$Range(w_{t})=Max(w_{t})-Min(w_{t})$$

### 2.2. Training

In the Training Phase, we investigate a wide range of machine learning algorithms to classify the training data, including Decision Tree, Random Forest, Support Vector Machine, K Nearest Neighbor, Logistic Regression, Naive Bayesian, and Neural Network. The classifiers are applied for recognition. The training data can be raw data from the sensors, or the features in different window lengths. The best classifier will be selected for indoors/outdoors detection.

### 2.3. Testing

In the testing phase, new samples are categorized using the classifiers created by the machine learning algorithms. Different algorithms perform differently. Researchers have proposed a lot of performance measures for multi-class classification.

Confusion matrix was proposed to evaluate the performance of a classification system for both 2-class and multi-class samples. Table 2 is a template of confusion matrix for a 3-class classifier.

In Table 2, the rows represent the true classes of the tested samples, and the columns represent the predicted classes. *n_ij_* is the number of test samples of a class *i* recognized as class *j*. Sometimes *n_ij_* would also be the percentage of the classification result. The nearer the confusion matrix is to a diagonal matrix, the better the classification algorithm is. Based on the confusion matrix, there are several widely used performance measures. Here we just introduce in Table 3 the ones we will use in the experiments.

## 3. Experiments

**Figure 2:**
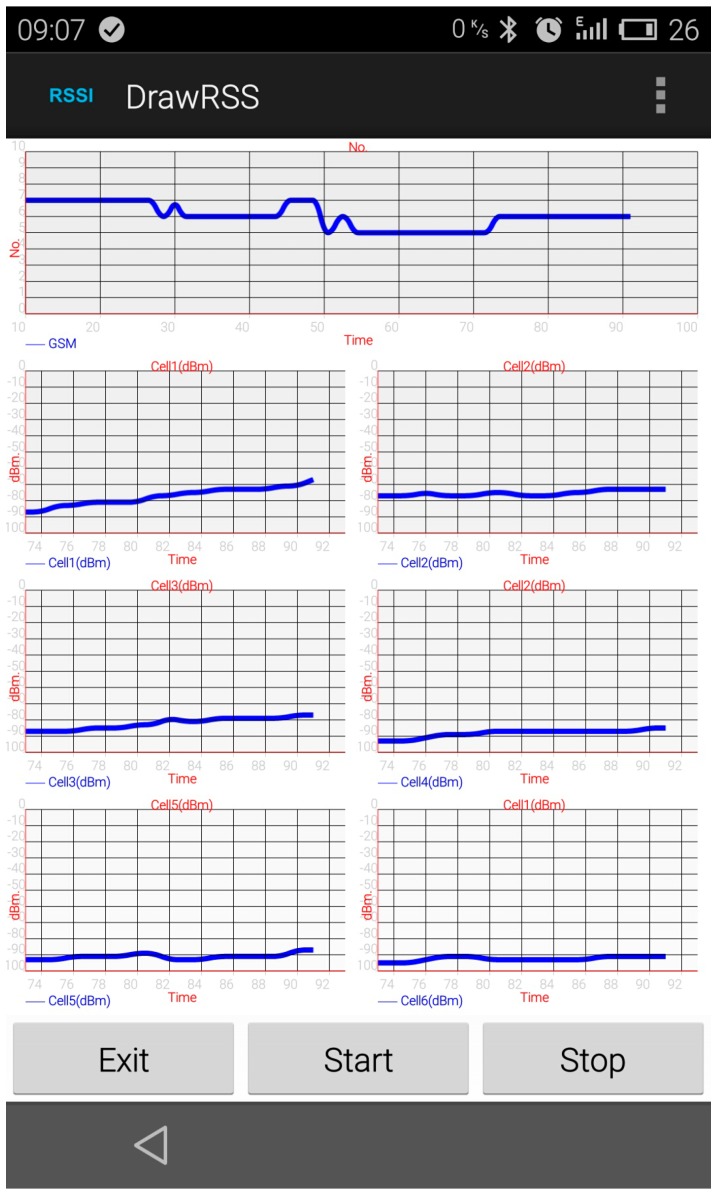
Screenshot of the DrawRSS application. In this application, all the neighboring cellular base stations’ signal strengths are drawn on the canvas and recorded in the phone’s memory.

To test the context sensing algorithm, we implemented an Android smart phone application capable of capturing nearby cellular base stations’ signal strength. The application is called DrawRSS. This application was tested on a Meizu MX3 smart phone (MeiZu, ZhuHai, China), which is running Android OS Version 4.4 (KitKat) (Google, Mountain View, CA, USA). A screen-shot of this application is shown in Figure 2.

**Figure 3:**
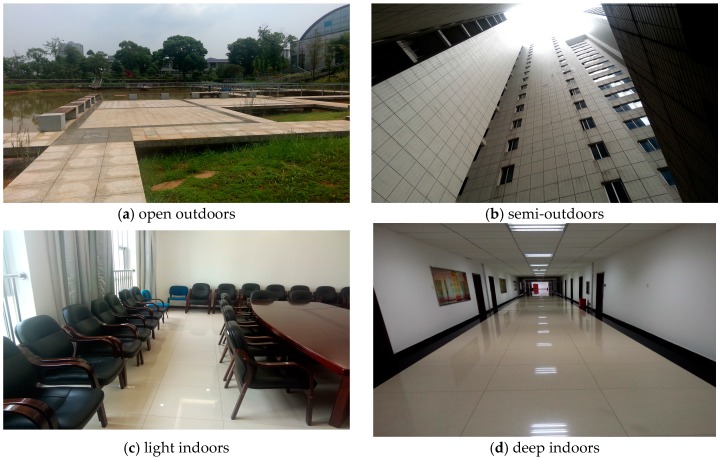
The four testing environments. These four environments are located on the same campus. The largest distance between them is less than 400 m.

The signal strength is recorded every 0.5 s, and then drawn oin the user interface, so the sampling rate is 2 Hz. The application records the number of cellular base stations and their signal strengths. The data are called training data. The testing environments in this experiment are shown in Figure 3.

During the experiment, the volunteer stayed in the four environments mentioned above for about 10 min. He could use his phone in the same manner he naturally would. He kept moving around in the environment. For example, in the light indoors environment, he walked in the room randomly. The training data are collected during these 10 min. In the deep indoors environment (Figure 3d), the volunteer moved from one side of the corridor to the other side several times.

**Figure 4:**
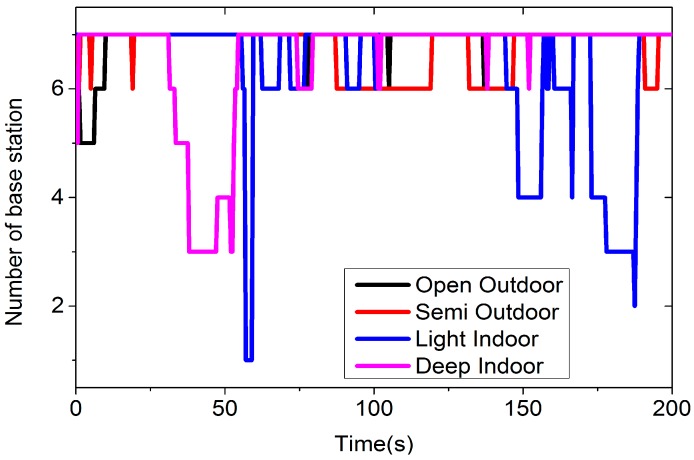
The number of cellular base stations in different environments.

Figure 4 is the number of cellular base stations in different environments recorded by the application. This figure only shows the result beyond the first 200 s.

**Figure 5:**
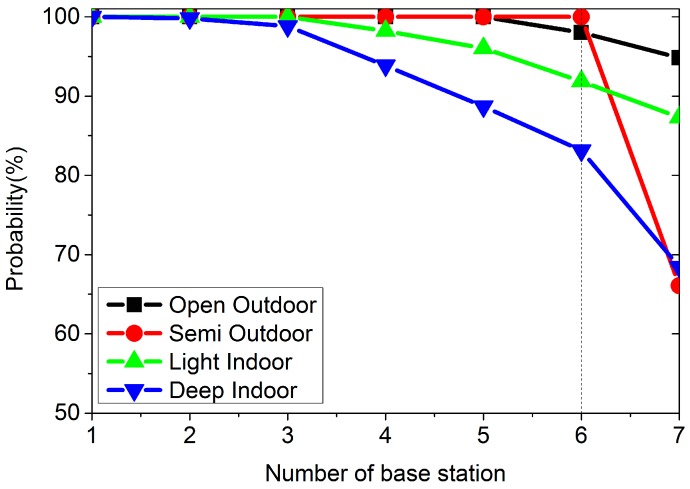
The probability curve of the number of GSM cellular base stations.

In Figure 4, we can see that the number of cellular base stations is quite different from the open outdoors to deep indoors environment. In the open outdoors environment, we have seven stations most of the time, while in the semi-outdoors environment, we often have six stations. In the indoors environment, the number of stations varies from 1 to 7. Figure 5 is the probability curves for different environments. Every node (*x*, *y*) in Figure 5 means there is *y*% probability of having at least *x* cellular base stations.

From Figure 5, we can see that we have at least one station in the specified four environments. If we want six cellular base stations, the probability is 98.02%, 100%, 91.87%, and 83.1% in the open outdoors, semi-outdoors, light indoors and deep indoors environments, separately. On average, the probability of having at least 1 to 7 base stations in any environment is 100%, 99.95%, 99.70%, 98.02%, 96.18%, 93.25%, and 79.17%, respectively.

**Figure 6:**
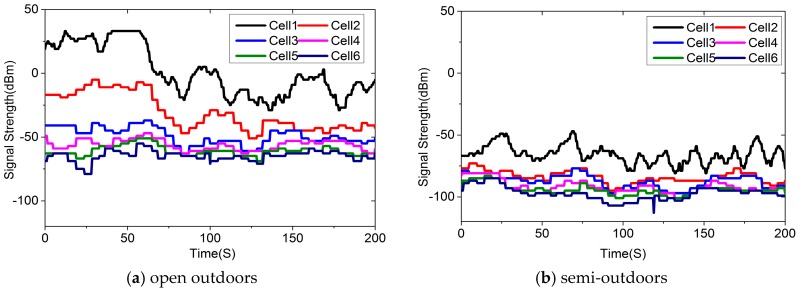

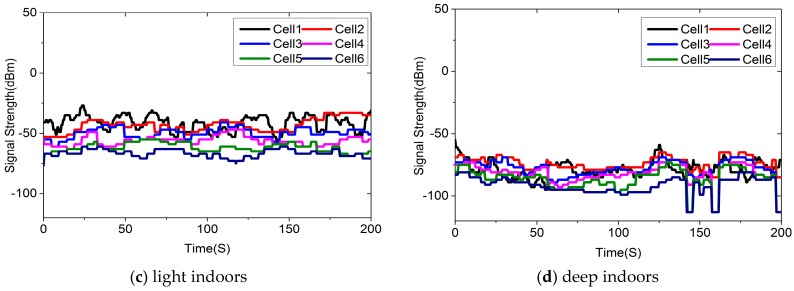
Six cellular base stations’ signal strength received in different environments.

We filtered out the samples with less than six cellular base stations and compare the signal strength received in the different environments in Figure 6. The widely used Log-Distance Path Loss (LDPL) signal propagation model [23] tells us that the signal strength is affected mainly by the distance. As a result, we will have the same signal strength measures in different environments. In Figure 6, we can find many examples, but if we take more stations’ signal strength into consideration, and we look at how the signal strength changes with time, we will find that different environments show different patterns. From Figure 6, we can see that the signal strength received in these four environments is quite different. These results imply that it is possible to identify different environments using the cellular base stations’ signal strength.

Before performing a classification using the raw data, we pre-process these data as mentioned above. The raw data are grouped according to different windows varies from 1 to 20 s. We calculate the features in each window, including Mean, Standard Deviation, Maximum, Minimum, and Range.

## 4. Data Analysis

The input data, including measured data and the pre-processed data are both ready for classification using the machine learning algorithms. In this section, we investigate the use of a wide range of machine learning algorithms to classify the training data, including Decision Tree, Random Forest, Support Vector Machine, K Nearest Neighbor, Logistic Regression, Naive Bayesian and Neural Network. The classifiers are applied for recognition. The classification performance will be compared between these Machine Learning algorithms.

**Figure 7:**
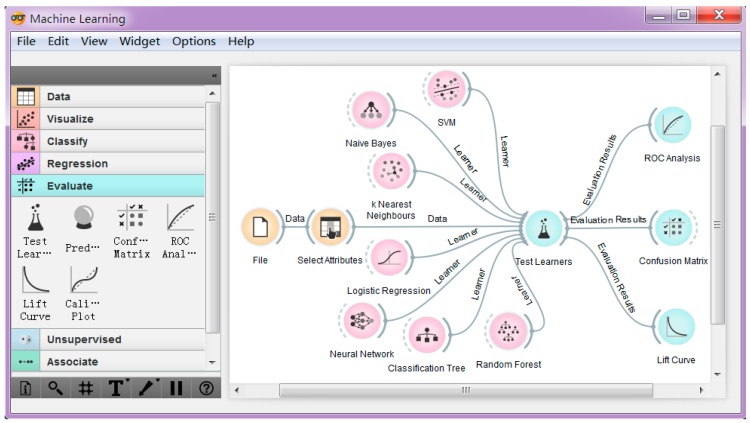
Workflow of the data classification using Orange toolkit.

In this paper, learning and classification is conducted by the Orange data mining toolkit [24]. Orange is an easy-to-use machine learning toolkit, which allows us to perform repeat model training using a wide range of machine learning algorithms and employing standard performance testing techniques, such as cross-validation without programming. Figure 7 shows the work flow of this experiment. 

**Figure 8:**
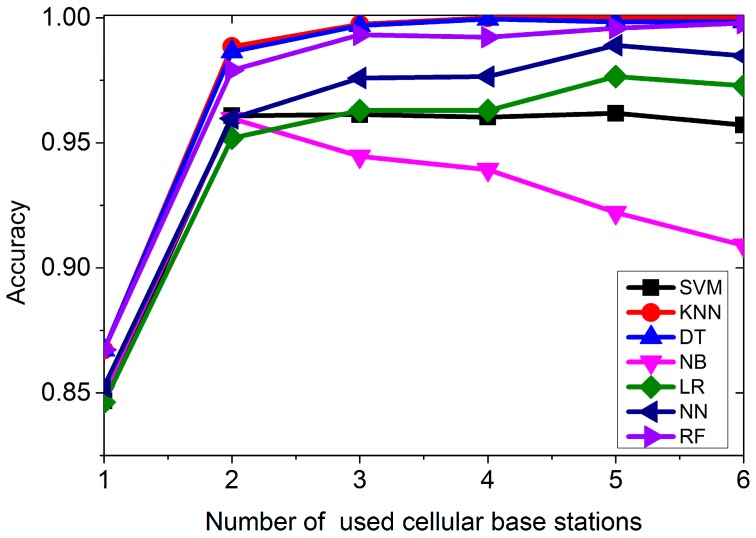
Classification accuracy using raw data.

In Figure 7, seven classification algorithms are created by dragging the corresponding widgets to the canvas. The file widget reads data from disk. Firstly, we apply the raw data for classification. The data are collected every 0.5 s in the four environments for 10 min, respectively. Figure 8 shows the classification accuracy. In Figure 8, we can see that for most of the classification algorithms, the more stations used, the better the accuracy is. KNN performs the best among the seven algorithms, followed by Decision Tree and Random Forest algorithm. Neural Network performs the worst. We just need four cells’ signal strength to get the best accuracy using KNN.

However, from Figure 5 we know that we cannot always receive signal from at least four stations. On average, the probability of receiving at least four cellular stations is 98.02% for all the cases. However, we will surely have at least one station in any case. In the following part of this section, we are going to using just one cellular base station’s signal strength for classification.

**Figure 9:**
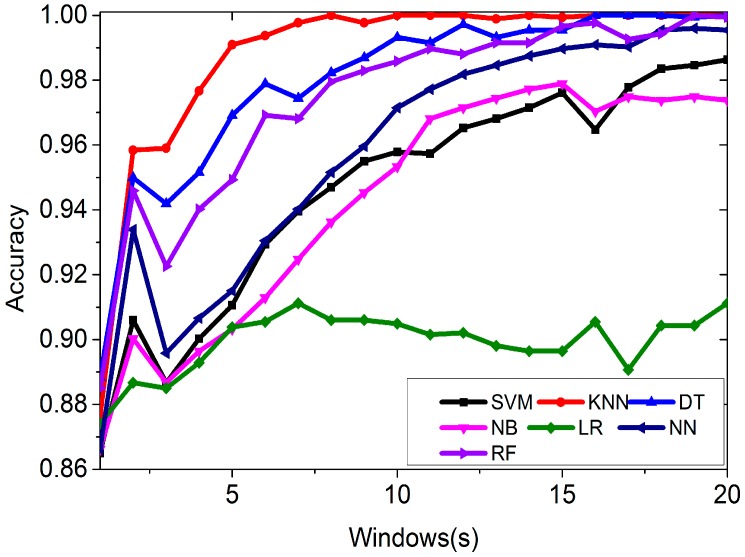
Classification accuracy using features varying with window length.

As mentioned above, we can calculate the features for different window lengths and apply the features for classification. The window length is varied from 1 to 20 s and we calculate the features in each window length, including Mean, Standard Deviation, Maximum, Minimum, and Range. In each window length, the number of instance is different. For example, there are 600 instances if the window length is 1 s. Figure 9 shows the accuracy of classifying the features.

From Figure 9, we can see that in most of the cases, the longer the window length is, the better the accuracy gets. Once again, KNN performs the best among all the algorithms, followed by the Decision Tree and Random Forest algorithms. Logistic Regression performs the worst. When the window length is 8 s, we can correctly classify all four environments using the KNN algorithm.

**Figure 10:**
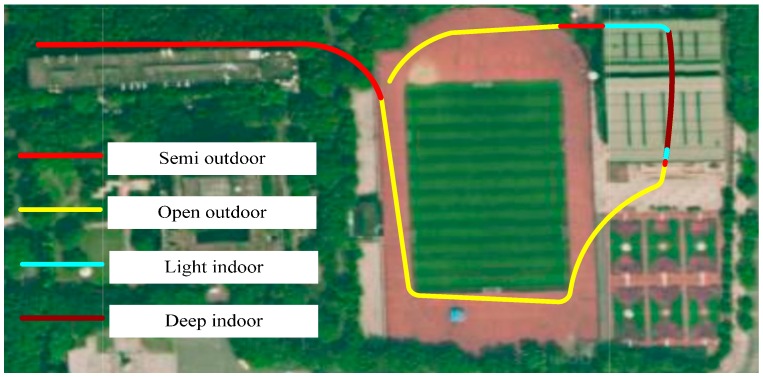
Testing trace located in the university campus. This trace contains two open outdoors segments, three semi-outdoors segments, two light indoors segments, and one deep indoors segment. The volunteer walks along the path in 10 min.

For testing this algorithm under different conditions, we experimented during one week with five different walking traces during the period from 9:00 to 17:00 under different weather conditions. These traces are different from the environments where we collected the data to generate the classifier. These walking traces contain nine open outdoors segments, 11 semi-outdoors segments, 10 light indoors segments, and 11 deep indoors segments. Figure 10 shows one of the walking traces that we experimented with.

In the experiments, the volunteer walks along this path while using his phone in the same manner he naturally would. The true environment type is manually labeled. Each day, these traces are tested three times in the morning, noon and afternoon, respectively.

**Figure 11:**
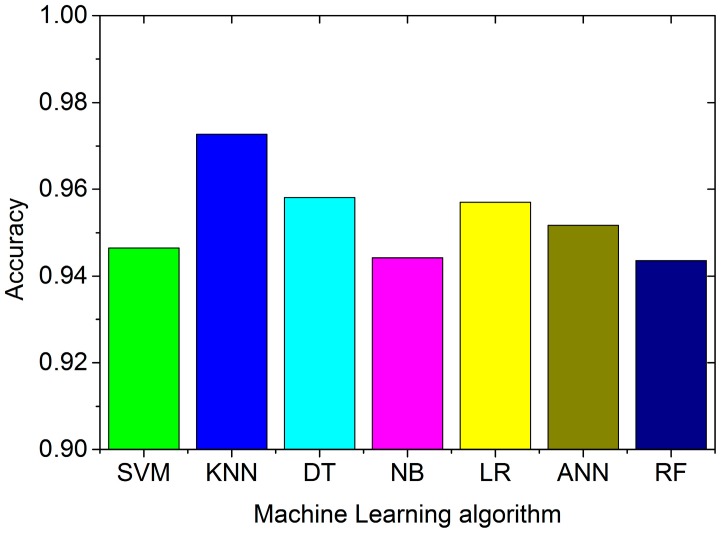
Classification accuracy using four stations’ signal strength with different classifiers created by different machine learning algorithms.

We first use the four cellular base stations’ signal strength for testing. The instances with less than four stations are filtered out. Figure 11 shows the average accuracy for different classifiers.

From Figure 11, we find that all the algorithms have accuracies better than 94%. KNN performs the best, with an average accuracy is 97.27%. Random Forest performs the worst, as its accuracy is 94.36%. The confusion matrix for KNN algorithm is shown in Table 4. The value *a_ij_* in this confusion matrix is not the number of the sample, but the percentage, given by the following expression:

*a_ij_* = *n_ij_*/*n_i_* × 100% (8)

Table 4 shows that we can detect open outdoors environments correctly. There is a 2.9% possibility of identifying deep indoors as light indoors environments, and a 1.4% possibility of identifying semi-outdoors as open outdoors environments. A light indoors environment might be classified as deep indoors with a possibility of 6.5%. However, we can’t always have at least four cellular stations. Figure 9 show that when the window length is 8 s, we can correctly classify all four environments using the KNN algorithm. In Table 5, we apply the different classifiers to detect the environment using 8 s window length.

From Table 5, we can see that Random Forest algorithm performs the best in all the measures. In the following comparison, we will apply the Random Forest algorithm for classification. Finally, we compare the proposed indoor/outdoor detection algorithm with the IODetector [2], Co-Training [3] and GPS based detection in terms of accuracy and energy consumption.

**Figure 12:**
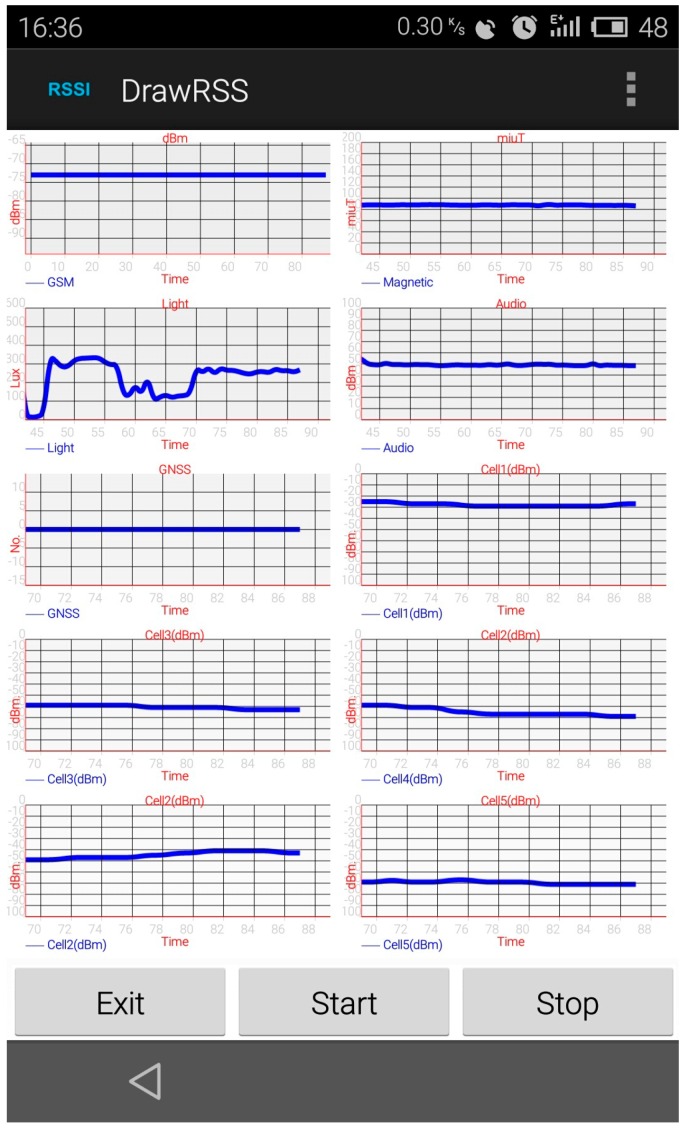
Screen shot of the updated application. The updated application records the light intensity, cellular signal strength, magnetic variance, sound intensity, and visible GPS satellites in the memory.

The app DrawRSS is updated to collect other required signal strengths, including light intensity, cellular signal strength, magnetic variance, sound intensity, and visible GPS satellites. A screen shot of the application is given in Figure 12.

We collect the required signal strength from the four environments for 10 min. The sampling rate is 2 Hz. The IODetector use the light intensity, cellular signal strength and magnetic variance to detect the indoor/outdoor environment. Co-Training uses the light intensity, cellular signal strength and sound intensity for detection. Our proposed algorithm uses the Random Forest algorithm to classify 8 s window length features from one cellular station’s signal strength. These four algorithms can detect different indoor/outdoor environments: co-Training and a GPS-based algorithm are proposed to detect indoor and outdoor environments, while IODetector is capable of detecting outdoors, semi-outdoors, and indoors; our proposed algorithm can detect four kind of different indoor/outdoor environment. In this experiment, all four of these algorithms only detect indoors and outdoors environments. Table 6 shows the results, confirming that the proposed algorithm performs the best among the four algorithms.

Different indoor/outdoor algorithms require different sensors. As a result, they consume different amounts of energy. According to [4], GPS consumes the most energy, followed by microphone, light sensor, and magnetic sensor. GSM consumes the least energy. Compared with the other indoor/outdoor detection algorithms, our proposed algorithm only needs the GSM sensor, which consumes minimal energy in addition to standard cell-phone operation, so the proposed algorithm is the most energy efficient among the four detection algorithms.

## 5. Discussion and Conclusions

Detecting the users’ current environment automatically is crucial for SNAL. In this paper we propose a GSM-based indoor/outdoor detection algorithm. Some studies [25,26] show that both the distance from the base station and the difference in a height of mobile phones influence the received signal strength. Our idea is to not just rely on the signal strength. We apply a machine learning algorithm to classify the neighboring GSM station’s signal in different environments, and identify the users’ current context by signal recognition. We test the algorithm in four different environments. The results show that the proposed algorithm is capable of identifying open outdoors, semi-outdoors, light indoors and deep indoors environments with 100% accuracy using four nearby GSM stations’ signal strength. Large scale experiments have proved the efficiency of the algorithm. The required hardware and signals are widely available in our daily lives, implying its high compatibility and availability. Future work will concentrate on real-time indoors/outdoors detection by introducing new low energy consumption signals that exist pervasively. The proposed approach was tested on only one phone. However, GSM RSSI may be different on different phones even for the same location. The device diversity problem is the one we must focus on in the future.

## Figures and Tables

**Table 1:** Four indoor/outdoor environments.

Environment	Open Outdoors	Semi-Outdoors	Light Indoors	Deep Indoors
Definition	Outside a building	Near a building	In a room with windows	In a room without windows
Example	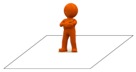	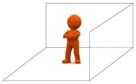	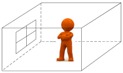	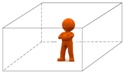

**Table 2:** Template of a confusion matrix for a 3-class classifier.

Class	Class 1	Class 2	Class 3
Class 1	*n*_11_	*n*_12_	*n*_13_
Class 2	*n*_21_	*n*_22_	*n*_23_
Class 3	*n*_31_	*n*_32_	*n*_33_

**Table 3:** Template of a confusion matrix for a 3-class classifier.

	Definition	Formula
True Positive (TP)	The number of samples of a class which have been correctly classified	$TP_{i}=n_{ii}$
True Negative (TN)	The number of samples of other classes which has been correctly classified	$TN_{i}=\sum_{j\neq i}\sum_{k\neq i}n_{jk}$
False Positive (FP)	The number of samples not belongs to a class which has been incorrectly classified as belonging to it	$FP_{i}=\sum_{k\neq i}n_{ki}$
False Negative (FN)	The number of samples belonging to a class which have been incorrectly classified as belong to other class	$FN_{i}=\sum_{k\neq i}n_{ik}$
Accuracy	The proportion of all samples which have been correctly classified	$Acc=\frac{\sum_{i}n_{ii}}{TP_{i}+TN_{i}+FP_{i}+FN_{i}}$
Sensitivity	The proportion of samples which have been correctly classified	$Sens_{i}=\frac{TP_{i}}{TP_{i}+FP_{i}}$
Precision	The proportion of sample predicted to belong to a class which is correct	$Prec_{i}=\frac{TP_{i}}{TP_{i}+FP_{i}}$
Specificity	The proportion of negative samples which have been correctly classified to be negative	$Spec_{i}=\frac{TN_{i}}{FP_{i}+TN_{i}}$
F-Measure	the weighted average of the precision and sensitivity	$F1=\frac{2}{1/Sens_{i}+1/Prec_{i}}$

**Table 4:** Confusion matrix using four stations’ signal strength with KNN classifier.

Environment	Deep Indoors	Semi-Outdoors	Light Indoors	Open Outdoors
**Deep Indoors**	97.1%	0	2.9%	0
**Semi Outdoors**	0	98.6%	0	1.4%
**Light Indoors**	6.5%	0	93.5%	0
**Open Outdoors**	0	0	0	100%

**Table 5:** Performance measures using 8 s window length with different algorithms.

Algorithm	Accuracy	Sensitivity	Specificity	F-Measure	Precision
**SVM**	0.8095	0.8095	0.9365	0.8077	0.8087
**KNN**	0.8652	0.8652	0.9551	0.8638	0.8634
**DT**	0.8861	0.8861	0.9621	0.8856	0.8901
**NB**	0.8734	0.8734	0.9578	0.8719	0.8717
**LR**	0.7994	0.7994	0.9331	0.7932	0.8051
**NN**	0.8677	0.8677	0.9559	0.8658	0.8652
**RF**	0.8943	0.8943	0.9647	0.8938	0.8933

**Table 6:** Accuracy comparison among the proposed algorithm, IODetector, Co-Training and GPS based detection.

	Random Forest	IODetector	Co-Training	GPS
Accuracy	95.3%	61.2%	93.14%	71.6%

## Supplementary Materials

The supplementary materials are available online at http://www.mdpi.com/1424-8220/16/10/1563/s1.
