# Supplementary figures and images for: Indoor-Outdoor Detection Using a Smart Phone Sensor
Source: Sensors (Basel). 2016 Sep 22;16(10):1563. doi: 10.3390/s16101563 (PMC5087352; doi:10.3390/s16101563)

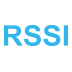

Supplement: Supplementary file 1 [file sensors-16-01563-s001.zip › Source Code/ContextSense02/bin/res/drawable-hdpi/ic_launcher.png]

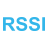

Supplement: Supplementary file 1 [file sensors-16-01563-s001.zip › Source Code/ContextSense02/bin/res/drawable-mdpi/ic_launcher.png]

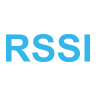

Supplement: Supplementary file 1 [file sensors-16-01563-s001.zip › Source Code/ContextSense02/bin/res/drawable-xhdpi/ic_launcher.png]

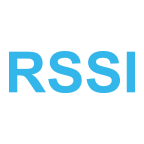

Supplement: Supplementary file 1 [file sensors-16-01563-s001.zip › Source Code/ContextSense02/bin/res/drawable-xxhdpi/ic_launcher.png]

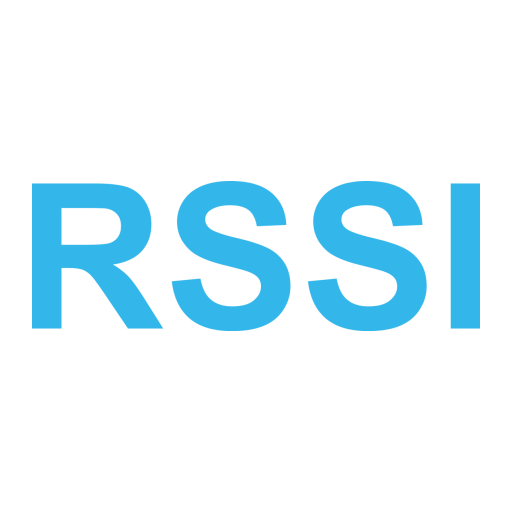

Supplement: Supplementary file 1 [file sensors-16-01563-s001.zip › Source Code/ContextSense02/ic_launcher-web.png]

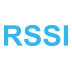

Supplement: Supplementary file 1 [file sensors-16-01563-s001.zip › Source Code/ContextSense02/res/drawable-hdpi/ic_launcher.png]

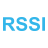

Supplement: Supplementary file 1 [file sensors-16-01563-s001.zip › Source Code/ContextSense02/res/drawable-mdpi/ic_launcher.png]

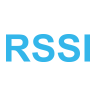

Supplement: Supplementary file 1 [file sensors-16-01563-s001.zip › Source Code/ContextSense02/res/drawable-xhdpi/ic_launcher.png]

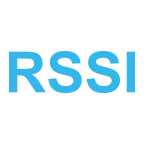

Supplement: Supplementary file 1 [file sensors-16-01563-s001.zip › Source Code/ContextSense02/res/drawable-xxhdpi/ic_launcher.png]
